# The Use of Mental Capacity and Mental Health Laws in the Care of People Living With Dementia in Residential and Hospital Settings: A Systematic Review of the Literature

**DOI:** 10.1177/14713012251367810

**Published:** 2025-08-15

**Authors:** Jeremy Dixon, Harry Bark, Chao Fang, Judy Laing, Demi Patsios

**Affiliations:** 1 Centre for Adult Social Care Research (CARE), Cardiff University, UK; 2 Department of Social & Policy Sciences, 1555 University of Bath, UK; 3 Department of Sociology, Social Policy and Criminology, 4591 University of Liverpool, UK; 4 University of Bristol Law School, 1980 University of Bristol, UK; 5 School for Policy Studies, 1980 University of Bristol, UK

**Keywords:** dementia, UNCRPD, mental capacity, mental health law

## Abstract

The UN Convention on the Rights of Persons with Disabilities (UNCRPD) has implications for the use of law with people living with dementia. This systematic review identifies how decisions to deprive people living with dementia of their liberty, using domestic mental capacity and mental health laws, are understood and made by professionals, and are experienced by people living with dementia and their carers. Database searches of Scopus, IBSS, CINAHL, PubMed, HMIC, and Social Policy and Practice were conducted to identify relevant academic and grey literature, with final searches in July 2024. No geographical or time scale restrictions were applied. Studies not written in English were excluded. Study quality was assessed using the Mixed Methods Appraisal Tool and thematic synthesis was used to organise data. The study was funded by Avon and Wiltshire Mental Health Partnership (grant number SP1200) and is registered on Prospero (CRD42023483046). Eleven studies were selected for review. Six papers were qualitative, three were quantitative and two employed mixed methods. Eight studies were based within the UK and all studies were conducted in Europe. Key themes were (1). Levels of legal understanding amongst stakeholder groups. (2). The influence of professional background on decision-making in dementia detention cases. (3). The impact of the severity of dementia and dementia diagnosis on decision-making. (4). The impact of care setting on decision-making. (5). The experiences of carers during decision-making. (6). The exclusion of people living with dementia during decision-making. Limitations are that not enough studies were identified to provide a meta-synthesis, non-English texts were excluded and that we did not specifically search for articles relating to guardianship laws. Our study identified significant gaps between the ideals of the UNCRPD and practice in health and social care settings.

## Introduction

In 2022, the United Nations’ Independent Expert on the Enjoyment of All Human Rights by Older Persons, highlighted the deprivation of liberty of older adults as a human rights concern (Mahler, 2022). The report identified a need to protect people living with dementia from unlawful deprivation in health and social care settings and from abuse within those settings (such as physical ill treatment, verbal abuse, or inappropriate forms of restraint). The United Nations Expert noted that the UN Convention on the Rights of Persons with Disabilities (UNCRPD) should form a key part of responding to such mistreatment. The UNCRPD is an international human rights framework which should be used to safeguard the rights of people with disabilities. Disability is not defined within the UNCRPD, but Article 1 refers to, “long term, physical, mental, intellectual or sensory impairments”, which would include dementia within its scope (United Nations, 2006). The UNCRPD has potential to strengthen human rights protection for persons with long term mental disabilities, including persons with dementia, as it includes positive rights and entitlements, such as the right to health (Article 25), in addition to civil and political rights, such as the right to liberty (Article 14). Although international treaties such as this are not directly enforceable in all nations, there is evidence that they exert influence on the development of national laws and policies (Fiala-Butora, 2024; Lang, 2021; O’Mahony, 2024).

Mental capacity laws and mental health laws vary internationally (Davidson et al., 2016; Saya et al., 2019). In this article, we define mental capacity laws as frameworks focussing on the decision-making competencies of individuals (which may authorise short or long-term detention), and mental health laws as frameworks which allow for people with mental health problems to be detained and treated compulsorily in defined circumstances. For example, in England and Wales, the (Mental Capacity Act, 2005) includes a presumption of capacity, but creates a framework of substituted decision-making for persons who lack capacity, and who are unable to make their own decisions, including decisions about their health and welfare, whilst the Mental Health Act (1983) (as amended) authorises detention and treatment on the basis of mental disorder coupled with risk to self or others. The UNCRPD has challenged state decision-making about the use of such laws in two ways. First, Article 12 of the Treaty states that persons with disabilities should enjoy ‘legal capacity’ (the formal ability to exercise rights and duties) on an equal basis with others. This has challenged state practices which treat mental capacity as a ‘threshold decision’ which allow for a professional to rule that a person lacks mental capacity based on factors such as their ability to process, recall or communicate information (Katona et al., 2009). Further, the Committee on the Rights of Persons with Disabilities (the UN body with responsibility for monitoring the UNCRPD) interpreted this to mean that all individuals should be judged to hold legal capacity and should be supported to decide for themselves (Committee on the Rights of Persons with Disabilities, 2014, para 28). Second, the UNCRPD identifies that state parties should ensure that people with disabilities, “[a]re not deprived of their liberty unlawfully or arbitrarily, and that any deprivation of liberty is in accordance with the law, and that the existence of a disability shall in no way justify a deprivation of liberty” (United Nations, 2006, Article 14, 1(b)). The Committee has interpreted this to mean, that no form of deprivation of liberty of people with disabilities is permitted in any context (Committee on the Rights of Persons with Disabilities, 2014, para 40–41). This interpretation signals that people living with dementia should be supported to decide for themselves and that neither substitute decision-making or a deprivation of liberty is lawful within care homes or hospitals. The interpretation creates challenges for state parties to effectively implement the CRPD fully at a domestic level, however, as it would effectively mean abolishing mental health and capacity laws (Bartlett, 2020, 2024). Consequently, the UNCRPD has generated considerable debate, with other UN treaty bodies adopting a different approach to deprivation of liberty on health grounds (United Nations, 2016) and some commentators calling for a more realistic interpretation of the convention (Dawson, 2015).

To date, much of the debate around the use of mental capacity and mental health laws for people living with dementia has remained academic in nature. It is therefore important to examine what the empirical evidence tells us. The aim of our systematic review is to identify how decisions to deprive people living with dementia of their liberty, using domestic mental capacity and mental health laws, are understood and made by professionals and are experienced by people living with dementia and their carers. Our research questions, which were registered on Prospero are:

(a) What processes are put in place to protect the legal rights of people living with dementia who are detained in health and social care settings?
(b) Who is involved in the decision-making process?
(c) How are the views of persons with dementia taken into consideration (or not)?
(d) To what extent are the views of family members considered throughout the detention process?
(e) What other factors impact on professional decision-making?

In considering question b, we focus on which professionals or workers were involved. In considering question e, we focus on decision-making in relation to the detention of people with dementia under domestic mental capacity laws or mental health laws.

## Methods

The team considered the aims and objectives of both scoping and systematic reviews (see, Munn et al., 2018). We chose to conduct a systematic review on the basis that we wished to uncover international evidence, identify current practice (and variations), inform areas for future research and to identify any conflicting results, whilst also reviewing the quality of current evidence. Our systematic review used the Prisma-P 2015 Checklist and drew on the Preferred Reporting Items for Systematic Review and Meta-Analyses (PRISMA) reporting guidelines (Moher et al., 2015) and is included in our Supplemental materials. Our research questions and search terms were informed by consultation with an advisory group made up of two people living with dementia, a carer, two mental health professionals and a senior academic. The protocol was registered on the PROSPERO database CRD42023483046) and can be accessed at https://www.crd.york.ac.uk/PROSPERO/display_record.php?RecordID=483046.

### Information Sources

After consultation with a librarian at the University of Bath the databases Scopus, IBSS, APA Pyschnet, CINAHL, PubMed and HMIC were used to identify papers. These databases were selected because of their broad focus on health and medicine, (including allied health and nursing) and humanities. Peer-reviewed and grey literature were searched for using Social Policy and Practice. We were aware that research studies on the use of mental capacity and mental health laws on people living with dementia were likely to be limited and so no geographical or time scale restrictions were applied to searches. For the same reason, we did not place any parameters around type(s) of dementia or other demographics, such as age, nationality or gender. As we had no funds for translation, we limited searches to English language publications.

### Search Strategy

The initial search was conducted by Author 2 and was cross-checked by Author 1. The search strategy was further informed following consultation with our advisory group. Our search terms were then refined, after a meeting with the subject librarian. We did not employ a validated search strategy tool. The following search terms were used and adapted for the various bibliographic databases, with Boolean operators being used when possible:

(dementia OR “alzheimer’s disease” OR “vascular dementia” OR (“lewy bodies” OR “lewy body”)) AND (decision-making OR decision*) AND (“Legal Rights” OR “legal framework*” OR “statutory framework*” OR “Deprivation of Liberty” OR Restriction OR “Restriction of Movement” OR “deprived liberty” OR “deprived of their liberty” OR Detention OR Detain*) AND (profession* OR famil* OR “service user” OR patient*).

We departed from our protocol through adding the search term “patient*” to the terms “profession* OR famil* OR “service user”. This change was made because the team felt that the term patient was likely to be used in preference to the term “service user” within the health literature. Pilot searches were trialled before registration, with further searches being conducted after registration and the final search being conducted in July 2024. We conducted forward and backward citation tracking using reference lists and the ‘Cited by’ function on Google Scholar.

### Condition being studied

Our study focussed on the use of mental capacity and mental health laws on people living with dementia in residential care or hospital settings. We included:

- Studies focussing on people living with dementia, their family members or health and social care workers.
- Studies focused on how professionals, people living with dementia or carers understand the use of mental capacity and mental health laws in relation to people living with dementia and their detention and how they are operationalised in hospitals, care homes or other residential care settings.

We excluded studies:

- Which did not consider the use of mental capacity or mental health laws or a deprivation of liberty.
- Which focussed on assessments relating to the use of mental capacity or mental health law or a deprivation of liberty but did not contain significant material related to dementia.
- Were not written in English.

### Data Management and Study Selection Process

Microsoft Excel spreadsheets were used to organise and manage references and abstracts. Duplicates were removed by Author 2. All titles and abstracts were screened against the eligibility criteria by two reviewers (with texts being distributed to Authors 1-3). In cases where disagreements arose, the reviewers met and reached a consensus decision. This process was repeated for full text screening. We did not record the number of agreements and disagreements amongst reviewers.

### Data Extraction

Data were extracted from the studies selected. Findings were extracted by all six researchers, in a word template, overseen by Author 1. The data extraction tool was developed though team discussion (see Supplemental materials). The tool recorded the column and reference ID (from screening), the citation and DOI, reviewer name, methods employed in the paper, and themes identified (in free text). Each study was reviewed by two research team members. The themes identified by reviewers were discussed within team meetings and any discrepancies were also discussed. If any disagreements remained, a third team member was asked to review, and themes were then discussed at a subsequent meeting.

### Quality Assessment

Study quality was assessed using the Mixed Methods Appraisal Tool (MMAT) (Hong et al., 2018). Authors 1-3 were involved in the quality assessment process. Two researchers applied the MMAT tool to each paper. The relevant MMAT quality assessment questions were applied, depending on the methodologies of the study, with ‘Yes’, ‘No’, or ‘Can’t Tell’ options for the quality assessment questions. It was agreed before screening that studies that scored two or more no/can’t tell responses on the methodological quality criteria would be excluded. No studies which were selected after abstract and full-text screening fell below this threshold and were excluded following quality assessment (Table 1).

**Table 1.** Quality Assessment Screening

Author	‘Yes’ Answer to one or both MMAT screening questions?	Meets relevant MMAT quality assessment criteria? (Views of two researchers)	Third opinion needed for consensus?	Final decision on inclusion/Exclusion based on MMAT quality assessment
Blasco et al. (2020)	Yes	2 x Yes	No	Include
Cairns et al. (2011)	Yes	2 x Yes	No	Include
Clare et al. (2013)	Yes	2 x Yes	No	Include
Emmett et al. (2013)	Yes	2 x Yes	No	Include
Gilburt (2021)	Yes	2 x Yes	No	Include
Lühnen et al. (2019)	Yes	2 x Yes	No	Include
Manthorpe et al. (2011)	Yes	2 x Yes	No	Include
Maxmin et al. (2009)	Yes	2 x Yes	No	Include
Paananen and Lindholm (2023)	Yes	2 x Yes	No	Include
Samsi et al. (2012)	Yes	2 x Yes	No	Include
Wolverson et al. (2023)	Yes	2 x Yes	No	Include

### Data Synthesis and Analysis

Thematic synthesis was used in which articles were coded to produce ‘descriptive’ and ‘analytic’ themes (Thomas & Harden, 2008). Author 1 and Author 2 independently conducted the data analysis following this process. The initial themes were then compared and refined collaboratively by all authors. When disagreements on themes arose, all authors engaged in discussion until a consensus was reached, ensuring the final thematic framework was both rigorous and reflective of the data. Quantitative data themes were generated to capture rights protection, decision-making dynamics, the inclusion of voices of people with dementia and families and factors influencing professional judgement. These themes were presented in narrative form as a meta-analysis could not be performed due to the heterogeneity of the data.

## Results

**Figure 1.**
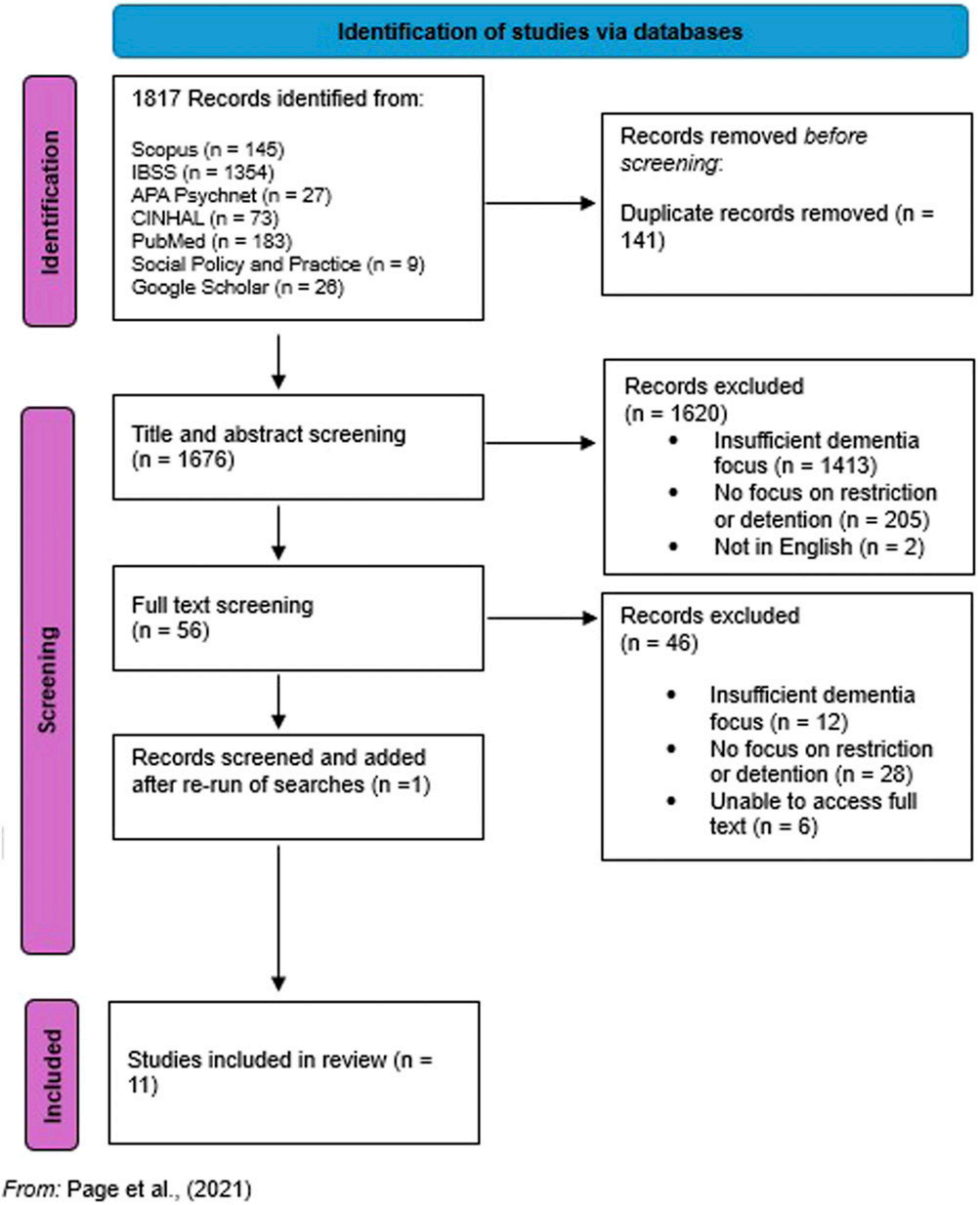
PRISMA Flow Diagram (Page et al., 2021)

Our systematic search and screening results are presented Figure 1. 1,817 papers were initially identified, which were reduced to 1,676 once duplicate papers had been removed. Following abstract, full-text screening and quality-review, 11 papers were identified as being in scope for our systematic review. No grey literature was selected. Whilst the use of mental capacity and mental health law relating to people living with dementia was of interest to policymakers and third sector organisations and documents were found which discussed these issues, no documents were identified which reported empirical data on topics within our inclusion criteria.

### Characteristics of Included Studies

Eleven studies were included in the systematic review (Blasco et al., 2020; Cairns et al., 2011; Clare et al., 2013; Emmett et al., 2013; Gilburt, 2021; Lühnen et al., 2019; Manthorpe et al., 2011; Maxmin et al., 2009; Paananen & Lindholm, 2023; Samsi et al., 2012; Wolverson et al., 2023). Six papers were qualitative, three were quantitative and two employed mixed methods. One study covered the whole of the UK, one study focused on England and Wales and six studies focused on England only. The other studies were also based in Europe with one study focussing on Finland, one Germany and one Spain. Included studies are summarised in Table 2.

**Table 2.** Study Characteristics

Author	Country	Design	Aims	Data collection methods	Sample	Data analysis methods
Blasco et al. (2020)	Spain	Quantitative	To appraise how physical and cognitive characteristics of nursing home residents were associated with use of restraint and to report on their prevalence	Retrospective cohort data collected about the type of restraint, the motivation for restraint and resident characteristics	Five publicly funded nursing home in Spain (each containing 120-140 residents)	Descriptive statistics
Cairns et al. (2011)	England	Quantitative	To compare the difference in decision-making between lawyers and mental health professionals when using the deprivation of liberty safeguards (mental capacity law) and the Mental Health Act	Professionals were asked to make a binary judgement about whether case information in a vignette amounted to a deprivation of liberty	Twenty-three professionals (6 lawyers, 17 mental health professionals)	Descriptive statistics
Clare et al. (2013)	England and Wales	Mixed methods	To investigate practitioner understanding of the deprivation of liberty safeguards and the Mental Health Act and to consider the effects of this interface on patients	Analysis of legal paperwork, comparison of the characteristics of people detained, semi-structured interviews with professionals, vignette study with psychiatrists	Twenty-nine professionals (interviews); 94 professionals (vignettes)	Legal paperwork analysed for patient characteristics; type of analysis for interviews and vignettes not stated
Emmett et al. (2013)	England	Qualitative	To investigate how assessments of capacity are made on people living with dementia on general hospital wards	Ethnographic ward observations, qualitative interviews with patients, nominated family members and professionals, informal conversations with ward staff and documentary analysis	Three ‘care of the elderly’ wards (acute and rehabilitation) in two hospitals	Comparative case study analysis
Gilburt (2021)	England	Mixed methods	To ascertain how the Mental Capacity Act and Mental Health Act are applied; which patient groups are most affected and in which settings decisions are made	Online survey to collect cross-sectional quantitative and qualitative data and 12 semi-structured interviews	Six hundred and thirty-four professionals (quant survey) and 12 professionals (qualitative interviews)	Descriptive statistics (quantitative data) Descriptive content analysis (qualitative data)
Lühnen et al. (2019)	Germany	Qualitative	To explore health decision-making processes with legal representatives of people living with dementia and to pilot test an information programme for this group	Interviews	Interviews with 12 professional representatives, 12 voluntary representatives and 14 senior citizens	Grounded theory
Manthorpe et al. (2011)	England	Qualitative	To assess staff ability to use mental capacity laws for people living with dementia in care homes	Interviews	Senior staff (17) and care workers (5)	Thematic analysis
Maxmin et al. (2009)	England	Quantitative	To assess the prevalence and predictors of mental capacity in older psychiatric patients (to make treatment and admission decisions)	Interviews using the MacArthur competency tool	Ninety-nine patients admitted to acute inpatient wards aged >65	Descriptive statistics, univariate and multivariate analysis
Paananen and Lindholm (2023)	Finland	Qualitative	To examine how nursing home staff discuss physical restrictions to family advocates	Care planning meetings between professionals and family members were audio or video recorded	Fifteen care planning meetings across six Finnish nursing homes; each containing 1-3 family members and 1-3 nursing staff members	Conversational analysis
Samsi et al. (2012)	England	Qualitative	To explore the experiences of community nurses about the Mental Capacity Act	Semi-structured interviews	Fifteen specialist dementia nurses	Thematic analysis
Wolverson et al. (2023)	United Kingdom	Qualitative	To investigate family carers experiences of inpatient mental health	Interviews	Seven family carers whose relative with dementia had been cared for within a mental health ward within the last five years	Reflexive thematic analysis

The predominant focus of studies was on staff understandings of mental capacity or mental health laws, with all these studies being focussed on England, or England and Wales. Three studies examined staff understanding of the Mental Capacity Act (2005), focussing on the views of care home managers and staff (Manthorpe et al., 2011), specialist dementia nurses (Samsi et al., 2012) and on health and social care professionals within hospital settings (Emmett et al., 2013). Three studies were concerned with the interface between mental capacity and mental health law; focussing on staff understandings and application of the (Mental Capacity Act, 2005) and Mental Health Act (1983) (Cairns et al., 2011; Clare et al., 2013; Gilburt, 2021).

The focus of the remaining papers was on the capacity of people living with dementia to make treatment or admission decisions (Maxmin et al., 2009); the way nursing staff justified the restraint of people living with dementia in care homes to family members (Paananen & Lindholm, 2023) and family experiences of their relative’s admission to a dementia inpatient ward (Wolverson et al., 2023). Most papers were concerned with the use of mental capacity or mental health laws in specific settings, with papers being focussed on deprivation of liberty/detention in hospital (Emmett et al., 2013; Maxmin et al., 2009; Wolverson et al., 2023), nursing homes (Blasco et al., 2020; Lühnen et al., 2019; Paananen & Lindholm, 2023), care homes (Manthorpe et al., 2011) and the community (Samsi et al., 2012). Tables 3 and 4 provide details of the included studies, including an overview of their design, themes, findings, limitations and recommendations (see Supplemental materials).

## Themes from Research Data

Following analysis of the data, six themes were identified which are reported below:

### Theme 1 - Levels of Legal Understanding Amongst Stakeholder Groups

A dominant theme throughout research papers was that stakeholder groups found mental capacity and mental health laws difficult to understand. Professionals struggled to apply mental capacity law to people living with dementia (Emmett et al., 2013; Manthorpe et al., 2011) and experienced it as difficult to communicate in layperson’s terms to people living with dementia and their family (Samsi et al., 2012). They also struggled with the interface between mental capacity or mental health law where a deprivation of liberty was being considered (Cairns et al., 2011; Gilburt, 2021). In some cases, practitioners were aware that other adult protection and human rights laws might apply but were uncertain as to which laws were relevant (Clare et al., 2013). A poor understanding of mental health law amongst family carers was also reported (Wolverson et al., 2023).

### Theme 2 - The Influence of Professional Background on Legal Decision-Making in Dementia Detention Cases

Three studies reported the impact of professional background on decision-making (Clare et al., 2013; Gilburt, 2021; Lühnen et al., 2019). A comparative study by Cairns et al. (2011) which examined how different professionals in England made judgements about deprivations-of-liberty found only slight agreement between lawyers, psychiatrists, best interests assessors and independent mental capacity advocates; including in cases of people living with dementia. Study authors reported that the difference in judgement is likely shaped by professional background with evidence showing that professionals favoured the laws they were most familiar with (Clare et al., 2013). Research also indicates a tendency to apply blanket rules about which types of law should be used in particular circumstances (Clare et al., 2013; Gilburt, 2021). Moreover, some legal principles, such as concepts of unwise decisions and mental capacity, within the Mental Capacity Act (2005) were understood by certain groups but not by others, reflecting variations in training and role-specific knowledge (Emmett et al., 2013).

Whilst professional background influenced decision-making, decisions were also affected by personal experiences and values with subjective values driving decision-making in some instances in matters relating to detention (Emmett et al., 2013; Lühnen et al., 2019). For example, in Emmett et al.’s (2013) study, the authors reported that nursing staff ignored the law or applied it selectively in order to reach what they perceived as the ‘right’ outcome for the person. Similarly, legal representatives in Lühnen et al.’s study (Lühnen et al., 2019) (court appointed volunteers or professionals) admitted that subjective criteria, such as personal convictions, empathy and sympathy held greater weight than the law when deciding on behalf of a person living with dementia.

### Theme 3 – The Impact of the Severity of Dementia and Diagnosis on Legal Decision-Making

Two papers highlighted that the severity of dementia had an impact on legal decision-making. One English study focussed on the prevalence and predictors of older adults (aged >65) to make capacitous decisions about treatment and hospital admission, of which people living with dementia made up 40.4% (*n* = 40) of the sample (Maxmin et al., 2009). It found that only one person living with dementia was able to make capacitous decisions about treatment, whilst around a quarter had capacity to make decisions relating to hospital admission. A further Spanish study, which studied types of restraint which were designed to restrict the freedom of movement of a person, observed that a diagnosis of dementia was one factor which led to nursing home staff recommending the use of restraints, with greater cognitive impairment being associated with more restraint, although some decisions were driven by the subjective values of professionals (Blasco et al., 2020).

One other article focussed on the impact of a diagnosis of dementia on professional practice. This English study found that a diagnosis impacted on the legal mechanisms which professionals recommended (Gilburt, 2021). In most cases, a diagnosis of dementia led staff to believe that the use of the Deprivation of Liberty Safeguards under the Mental Capacity Act (2005) was most appropriate, whilst a diagnosis of dementia alongside a functional mental illness, neurodevelopment or neurocognitive condition, led decision-makers towards using the Mental Health Act (1983).

### Theme 4 – The Impact of Care Setting on Decision-Making

Two articles reported that the place at which an assessment of whether a person with dementia met the legal criteria for detention under mental capacity or mental health law took place had an impact on legal decision-making. Interview and survey responses in an English study found that professionals often applied blanket rules to places of care (Gilburt, 2021). Decision-makers in this study felt that the use of deprivation-of-liberty safeguards under the Mental Capacity Act (2005) was most appropriate in settings that predominantly provided care for people with physical health problems, such as residential care homes. Conversely, the Mental Health Act (1983) was seen to be most appropriate where individuals were placed within a psychiatric hospital. Similarly, a German study noted that environmental factors were seen by professionals to impact on decision-making, although the way it did so was not stipulated (Lühnen et al., 2019).

### Theme 5 – The Experiences of Carers during Legal Decision-Making

Two research papers focussing on professionals’ perspectives stated that the views of family were considered during legal decision-making (Clare et al., 2013; Manthorpe et al., 2011). For example, professionals were more likely to view treatment in hospital as a deprivation of liberty where family members had expressed a wish for them to be treated at home (Clare et al., 2013). However, two qualitative studies highlighted practices in which carers were excluded from decisions (Paananen & Lindholm, 2023; Wolverson et al., 2023). Observations of care planning meetings in Sweden found that care home staff informed families about restraint which had already occurred and sought their approval afterwards (Paananen & Lindholm, 2023). Similarly, interviews with family members in the United Kingdom identified that they felt uninformed by staff about the legal frameworks used for detention (Wolverson et al., 2023). Family members felt that their views were ignored throughout the admission process and were left unclear about when an assessment for detention under mental health law might take place or how long detention would last.

### Theme 6 – The Exclusion of People Living with Dementia within Legal Decision-Making

A final theme from the research was that the views of people living with dementia were overlooked. Legal representatives in a German study reported that services commonly failed to involve people living with dementia in legal decisions, including those around decision-making capacity (Lühnen et al., 2019). A similar lack of involvement was noted in a Swedish study observing care planning meetings discussing use of restraints (Paananen & Lindholm, 2023). The researchers observed that people living with dementia were only present at three out of 15 meetings, with the person being absent or asleep during discussions around restraint in two of these. Notably, no studies reported on the views of people living with dementia under mental capacity or mental health law.

## Discussion

Our study has identified significant gaps between the ideals of the UNCRPD and practice in health and social care settings, related to deprivation of liberty decisions for persons living with dementia, especially in the context of Article 12 and Article 14.

Our systematic review indicates that professionals and carers found mental capacity and mental health laws difficult to understand. Whilst this was a dominant theme, these finding came exclusively from studies from the United Kingdom (specifically; England, Northern Ireland and Wales). This may reflect the complexity of laws in these jurisdictions, where criticism has been made about the dividing line between mental capacity and mental health legislation in England and Wales (Independent Review of the Mental Health Act, 2018). Specifically, it remains unclear whether the (Mental Capacity Act, 2005) or the Mental Health Act (1983) should be viewed as ‘less restrictive’ and in what circumstances they should be used, with Government promises to clarify the law being repeatedly postponed (see, Series, 2025).

Several studies highlighted discrepancies in legal decision-making within and between professional groups. Findings indicate that levels of agreement amongst legal professionals was low (Cairns et al., 2011). Such discrepancies might occur because lawyers lack confidence in understanding the nature and effects of dementia, as evidenced by research within this review (Lühnen et al., 2019). Furthermore, where a person living with dementia has lost capacity, legal professionals may feel unable to consider the views and wishes of the person, on the basis that they are unable to give legal instructions, as indicated in research with legal professionals in Australia (Sinclair et al., 2021). Different explanations may be given for the lack of consistency between professional groups. Variable levels of legal knowledge amongst professional groups may account for some differences (Peay et al., 2001). However, it is unlikely that this provides a full explanation. Professional groups are influenced by their own codes and guidelines and tend to view legal principles through the lens of their own profession, identifying ways that legal principles may be interpreted within their existing roles and boundaries (Castles, 2018). Additionally, groups vary in the extent to which human rights frameworks are foregrounded (Sinclair et al., 2021). Differences may also exist in the way that diverse groups interpret law and policy to balance harms which people living with dementia may experience (such as loss of autonomy) against risk to themselves or others (Peay, 2003; Stevenson & Taylor, 2017).

Research which investigates the impact of the severity of dementia or compares variability amongst those with different diagnoses is sparse. The paper by Maxmin et al., (2009) indicates that people living with dementia are less likely to be able to make capacitous decisions around treatment or hospital admission, but has a relatively small sample, with people living with dementia being a sub-group, rather than the focus of the study. Our review also highlights a paucity of research focussed on how the severity of dementia or dementia diagnoses impact on legal decision-making. Whilst the paper by Blasco et al. (2020) indicates that staff decisions around restraint are associated with the perceived severity of dementia, the research does not focus on the rationale for restraint use; a limitation which has been reported in research focussed on the use of restraint for people living with dementia more generally (Pu & Moyle, 2022). Whilst the paper by Gilburt (2021) suggests that diagnosis does have an impact on legal decision-making, greater research in this area is needed.

The need for carer engagement and involvement where mental capacity and mental health laws are used has been acknowledged for some time (Larkin et al., 2022). Our review highlighted sharp disparities in professional and carer perspectives on the issue. On the one hand, professionals in the studies we have reviewed report that they seek to balance the needs and wishes of people living with dementia and their carers. On the other hand, observations of practice and interviews with carers suggest that carer consultation remains tokenistic; which mirror findings with carers in adult mental health settings (Dixon et al., 2022; Wormdahl et al., 2021). As with other areas in our review, the number of studies remains limited, indicating that caution should be applied to current findings. However, it is important to establish how far families are involved in decisions around the use of mental capacity and mental health laws as research indicates that family members remain key to supported decision-making from the perspective of people living with dementia (Sinclair et al., 2019). This is because family members can provide decision-making support in efficient ways, due to their existing relationship with the person.

A key claim by dementia activists is that there should be “nothing about us without us”. Drawing on this phrase, disability researchers have highlighted that dementia researchers have historically excluded the views of people living with dementia, focusing instead on the views of carers or proxy decision-makers and have argued that this needs to change (Oldfield, 2021). Despite such demands, only one of the articles in our systematic review focussed directly on people living with dementia themselves and their ability to make capacitous decisions (Maxmin et al., 2009). Notably, there was a complete absence of qualitative research on the views of people living with dementia towards mental capacity or mental health law. This is a significant omission given the obligation placed on states to involve persons with mental disabilities in law, policy and decision-making under Article 4(3) of the UNCRPD.

Our systematic review highlights several priorities for research. Whilst several studies have focused on professional decision-making, greater empirical work in this area would be welcomed given that there is a lack of agreement amongst governments as to how the legal rights of people living with dementia should be protected where dementia is severe and the person loses the ability to participate, even with support (Scerri, 2016). Notably research studies in this area are absent outside of Finland, Germany, Spain and the UK, although due to our focus on studies in English some studies may have been missed.

A broader research base is needed to guide global knowledge and understanding given the projected incidence of dementia to 150 million people by 2050 (World Health Organization, 2017). The promotion of a toolkit which encourages the inclusion of people lacking capacity to consent to research, has the potential to increase the number of studies focussed on the detention of people living with dementia in care settings (Shepherd et al., 2024). The lack of studies focussed on carer perspectives identifies the need for more research of this type, particularly as family caregivers are often given an advocacy role in protecting and negotiating the best interests of people with dementia – a role that is often difficult to exercise (Emmett et al., 2014). Further research should also focus on the knowledge and perspectives of people living with dementia. Drawing on previously tested methodologies, which have focussed on older adults’ knowledge of their legal rights (Doron & Werner, 2008), researchers might develop a survey to test people living with dementia’s awareness around mental health and capacity law. There is scope for studies to identify ways to support capacitous decision-making in people living with dementia, in line with recent studies promoting supported decision-making more generally (Arstein-Kerslake et al., 2017).

Our findings raise several implications for practice. Both legal professionals and family-members may be called upon to advocate for people living with dementia under legal systems. However, there is no guarantee that either group will have a good understanding of dementia prognosis or treatment or of the relevant laws and human rights principles. Our review shows that professionals and the public often have low levels of understanding in relation to mental capacity and mental health laws, echoing findings from other studies (Dixon et al., 2020; Shepherd et al., 2018; Sullivan et al., 2023). This identifies the need for training programmes which have the potential to improve the mental health and legal literacy of such groups (Laing et al., 2024; Lühnen et al., 2019).

Several limitations should be noted of this review. As laws and policies vary across jurisdictions and studies were limited, it was not possible to provide a meta-synthesis. Studies not written in English were excluded meaning that data from other countries may be missing. Additionally, other jurisdictions have guardianship laws which allow for substitute decision-making. We searched for material related to legal rights, legal frameworks and statutory frameworks, but did not specifically search for articles focussed on guardianship processes. Consequently, it is possible that some processes related to the deprivation of liberty or restriction of movement of people living with dementia may have been missed.

In conclusion, it has been claimed that the UNCRPD represents a paradigm shift for people living with dementia (Shakespeare et al., 2019). Our systematic review of research focussing on how decisions to deprive people living with dementia of their liberty using mental capacity and mental health laws are understood and made by professionals, and understood by people living with dementia and carers, indicates that empirical evidence is limited. Furthermore, the available evidence suggests that principles of the UNCRPD are not being enforced in practice, which may relate to the debates about its interpretation and utility, mentioned in our introduction. Levels of legal understanding remain low across stakeholder groups and differences exist in the application of legal principles amongst professional groups. The severity and type of diagnosis as well as the type of care setting appears to have an impact. There is little evidence of people living with dementia and their carers being central to decision-making, as the UNCRPD suggests. Our review indicates the need for greater research into the use of mental capacity and mental health laws for this group as well as the need for validated training tools for legal decision-makers.

## Supplemental Material


Supplemental Material - The Use of Mental Capacity and Mental Health Laws in the Care of People Living With Dementia in Residential and Hospital Settings: A Systematic Review of the Literature
Supplemental Material for The Use of Mental Capacity and Mental Health Laws in the Care of People Living With Dementia in Residential and Hospital Settings: A Systematic Review of the Literature by Jeremy Dixon, Harry Bark, Chao Fang, Judy Laing, Demi Patsios in Dementia

## Data Availability

This article is a systematic review. The protocol was registered on the PROSPERO database (CRD42023483046) and can be accessed at https://www.crd.york.ac.uk/PROSPERO/display_record.php?RecordID=483046.
