# Supplementary material for: The Use of Mental Capacity and Mental Health Laws in the Care of People Living With Dementia in Residential and Hospital Settings: A Systematic Review of the Literature
Source: Dementia (London). 2025 Aug 15;25(3):654–73. doi: 10.1177/14713012251367810 (PMC13002962; doi:10.1177/14713012251367810)
Supplement: Supplemental Material - The Use of Mental Capacity and Mental Health Laws in the Care of People Living With Dementia in Residential and Hospital Settings: A Systematic Review of the Literature [file sj-pdf-1-dem-10.1177_14713012251367810.pdf]

Table 3 – Findings from qualitative and mixed methods studies

Author	Design	Key themes relating to detention under mental health or mental capacity law for people living with dementia	Executive summary of findings	Limitations	Recommen-dations
Clare et al (2013).	Mixed methods.	People living with dementia are one group who may 'fall between the gaps' within Deprivation of Liberty Safeguards. This relates to compliant informal patients whose lack of capacity to give consent goes unnoticed in either psychiatric hospitals or in general hospitals.	Practitioners gave preference to the Mental Health Act in psychiatric hospitals and the Deprivation of Liberty Procedures in 'care settings'.	None listed. However, some primary professional roles have little or no representation in the interviews and vignettes study.	i) Strengthen attention to decision-making capacity in all hospitals ii) Revise statutory paperwork iii) Revise Government legal guidance iv) Revise and improve data monitoring procedures.
Emmett et al (2013).	Qualitative.	Different professionals varied in their approach to applying the Mental Capacity Act, using either a 'functional' approach (applying the law as intended) or an 'outcome-driven' approach (in which decisions were based on their own values, in contravention to the law).	Legal standards are not routinely applied when decisions are made in relation to discharge following hospital admission.	Not specifically stated but observations were limited to three wards in one part of England.	A more specific test of mental capacity should be adopted when assessing whether a person is fit for discharge.
Gilburt (2021).	Mixed methods.	Interview participants indicated that they viewed the Mental Health Act as the most appropriate framework where dementia and mental illness existed and the Mental Capacity Act where dementia alone	Several characteristics affected whether practitioners used mental health law or mental capacity law including i) type of illness ii) organizational setting iii) treatment and care required by the patient iv)	Survey completed by a self-selecting group of practitioners; no judgement was made about the quality of practice; free text responses within the survey allowed participants limited space to explore	i) Mental health and mental capacity law should be reviewed so that alignment and simplification can be considered ii) safeguarding measures to ensure that the law is not misused should be reviewed to reduce unlawful deprivations of

		was indicated.	patient presentation v) transcience of capacity.	decisions.	liberty.
Lühnen et al (2019).	Qualitative.	Decision-making around physical restraint by legal decision makers was highly influenced by their subjective values.	The attitudes of voluntary representatives, professional representatives and senior citizens towards the use of physical restraint were heterogeneous.	Transcript and analysis results not returned to participants for comments; no people living with dementia could be recruited for interview.	The PRODECIDE education programme for improving legal decision-making appears feasible but should be tested through a randomized control trial.
Manthorpe et al (2011).	Qualitative.	Lack of knowledge amongst care workers and managers regarding the Mental Capacity Act (including Deprivation of Liberty Safeguards).	The care workers interviewed were generally not aware of parts of mental capacity law and managers were also about definitions for a deprivation of liberty.	Sample may be biased by voluntary participation; difficulty assessing the honesty of participants' responses.	Importance of providing regular legal training across different levels of the organization in care homes.
Paananen and Lindholm (2023).	Qualitative.	Staff accounts of restraint i) highlight threats to residents should restraint not be used ii) identify the benefits of restraint.	Nurses outline the process of restriction to relatives before highlighting the benefits of restraint and seeking agreement from families (discouraging dissent).	Small sample size (six nursing homes); research conducted during the Covid pandemic which limited observations and may have affected practice.	Family members should be i) involved in decision-making about restraint at an early stage ii) protocols within care plan meetings should be adjusted to promote family advocacy.
Samsi et al (2012).	Qualitative.	Participants felt that the Mental Capacity Act 2005 empowered dementia nurses.	Participants believed that the Mental Capacity Act 2005 gave dementia nurses greater powers to make best interests decisions in the community which could reduce levels of detention.	The study is focused on a small group of specialist dementia nurses; it is difficult to ascertain the accuracy of practitioner reflections and practitioners may have reported positive experiences of the law to appear knowledgeable.	Specialist nurses need greater knowledge of mental capacity laws to support service users and carers.
Wolverson et al (2023).	Qualitative.	i) The traumatic events leading to admission and how admission added to this distress ii) feeling	Family members were exhausted by caring at the point of admission, but the manner of	Small exploratory study with a sample size of seven.	Carers should be offered therapeutic support following admission and wards should not assume family

		excluded from professional decision-making around admission iii) lack of support for family at point of admission.	admission added to their distress. Family members experienced feelings of grief and guilt and were also concerned about the impact of admission on their relative.		members understand mental health law.

Table 4 – Findings from mixed methods and quantitative studies

**Author**	**Design**	**Key outcomes relating to detention under mental health or mental capacity law for people living with dementia**	**Executive summary of findings**	**Limitations**	**Recommendations**
Blasco et al (2020)	Quantitative	Descriptive statistics of socio-demographic variables and physical/cognitive assessments. Self-organized maps used for clustering residents.	Residents with greater cognitive impairment and functional limitations are more likely to be restrained. Subjective factors are significant. Self-organized map analysis showed three resident profiles associated with use of restraint.	Retrospective design may introduce biases; reported loss of observations, an exclusion of bedrails and a failure to consider residents' clinical conditions could have led to an underestimation of restraint.	i) Minimize influence of subjective factors in decision-making. ii). promote assessment of risk factors/individual needs iii) develop clear definitions of physical restraints, along with laws outlining alternatives.
Cairns et al (2011)	Quantitative	Descriptive statistics, Kappa coefficient values (to assess interrater reliability), and logistic regression analysis used to compare judgments about Deprivation-of-Liberty made by mental health professionals and lawyers.	Disagreement among professionals about Deprivation-of-Liberty assessments resulting in less reproducible and reliable outcomes.	Statistical power limited by small sample size; incomplete data collection for some vignettes may have impacted level of agreement.	i) Need better legal guidance on what constitutes a Deprivation-of-Liberty and how it should be assessed ii) training for clinicians iii) research into the validity of training tools.
Clare et al (2013)	Mixed methods	Deprivation-of-Liberty Safeguards data under the Mental Capacity Act and Mental Health Minimum Dataset combined	Most Deprivation-of-Liberty applications involved people living with dementia. A lack of understanding regarding the	See Table 2	See Table 2

		to identify overlaps/ differences in the characteristics of individuals subject to mental health and mental capacity law.	interface between Deprivation-of-Liberty Safeguards and the Mental Health Act due to differences in principles and criteria between the two Acts.		
Gilburt (2021)	Mixed methods	Online survey capturing decision-making factors of professionals involved in assessing people under the Mental Health Act or Mental Capacity Act. Descriptive statistics on health and mental capacity legislation in practitioners' understanding and decision-making factors.	Decision-making in psychiatric hospitals favors the Mental Health Act over Mental Capacity Act. Mental Capacity Act Deprivation-of-Liberty Safeguards are used for detaining people receiving care in psychiatric hospitals. Lack of understanding at the interface between the Mental Capacity Act and Mental Health Act.	See Table 2	See Table 2
Maxmin et al (2009)	Quantitative	Descriptive statistics tested for associations between patients with and without capacity to make decisions. Logistic regression identified predictors of mental capacity to make treatment and admission decisions. Inter-rater reliability assessed through joint interviews and consensus meetings.	An individual may lack capacity to decide in one area while retaining capacity in others. Lack of capacity was strongly related to dementia diagnosis. Predictors of lacking capacity were a lower Mini Mental State Examination score and less insight. Patients without capacity usually wanted doctors to make decisions for them, rather than family members.	No measure of symptom severity or psychopathology included. MacCAT-T is difficult to use to assess capacity for admission decisions. Did not exclude patients without a capacity to agree to the research, which may have led to an overestimation of the prevalence of capacity.	Tools are needed that consider nonverbal information and simple, non-technical language to aid in the assessment of capacity in moderate to severe cognitive impairment.

PRISMA 2020 Checklist

Section and Topic	Item #	Checklist item	Location where item is reported
**TITLE**			
Title	1	Identify the report as a systematic review.	Title
**ABSTRACT**			
Abstract	2	See the PRISMA 2020 for Abstracts checklist.	Abstract
**INTRODUCTION**			
Rationale	3	Describe the rationale for the review in the context of existing knowledge.	Introduction
Objectives	4	Provide an explicit statement of the objective(s) or question(s) the review addresses.	Introduction
**METHODS**			
Eligibility criteria	5	Specify the inclusion and exclusion criteria for the review and how studies were grouped for the syntheses.	Methods
Information sources	6	Specify all databases, registers, websites, organisations, reference lists and other sources searched or consulted to identify studies. Specify the date when each source was last searched or consulted.	Methods
Search strategy	7	Present the full search strategies for all databases, registers and websites, including any filters and limits used.	Methods
Selection process	8	Specify the methods used to decide whether a study met the inclusion criteria of the review, including how many reviewers screened each record and each report retrieved, whether they worked independently, and if applicable, details of automation tools used in the process.	Methods
Data collection process	9	Specify the methods used to collect data from reports, including how many reviewers collected data from each report, whether they worked independently, any processes for obtaining or confirming data from study investigators, and if applicable, details of automation tools used in the process.	Methods
Data items	10a	List and define all outcomes for which data were sought. Specify whether all results that were compatible with each outcome domain in each study were sought (e.g. for all measures, time points, analyses), and if not, the methods used to decide which results to collect.	Methods
	10b	List and define all other variables for which data were sought (e.g. participant and intervention characteristics, funding sources). Describe any assumptions made about any missing or unclear information.	Methods
Study risk of bias assessment	11	Specify the methods used to assess risk of bias in the included studies, including details of the tool(s) used, how many reviewers assessed each study and whether they worked independently, and if applicable, details of automation tools used in the process.	Methods
Effect measures	12	Specify for each outcome the effect measure(s) (e.g. risk ratio, mean difference) used in the synthesis or presentation of results.	Methods
Synthesis methods	13a	Describe the processes used to decide which studies were eligible for each synthesis (e.g. tabulating the study intervention characteristics and comparing against the planned groups for each synthesis (item #5)).	Methods
	13b	Describe any methods required to prepare the data for presentation or synthesis, such as handling of missing summary statistics, or data conversions.	Methods
	13c	Describe any methods used to tabulate or visually display results of individual studies and syntheses.	Methods
	13d	Describe any methods used to synthesize results and provide a rationale for the choice(s). If meta-analysis was performed, describe the model(s), method(s) to identify the presence and extent of statistical heterogeneity, and software package(s) used.	Methods
	13e	Describe any methods used to explore possible causes of heterogeneity among study results (e.g. subgroup analysis, meta-regression).	Methods
	13f	Describe any sensitivity analyses conducted to assess robustness of the synthesized results.	Methods
Reporting bias assessment	14	Describe any methods used to assess risk of bias due to missing results in a synthesis (arising from reporting biases).	Methods
Certainty assessment	15	Describe any methods used to assess certainty (or confidence) in the body of evidence for an outcome.	Methods

PRISMA 2020 Checklist

Section and Topic	Item #	Checklist item	Location where item is reported
**RESULTS**			
Study selection	16a	Describe the results of the search and selection process, from the number of records identified in the search to the number of studies included in the review, ideally using a flow diagram.	Results, Figure 1
	16b	Cite studies that might appear to meet the inclusion criteria, but which were excluded, and explain why they were excluded.	Results
Study characteristics	17	Cite each included study and present its characteristics.	Results, Table 2
Risk of bias in studies	18	Present assessments of risk of bias for each included study.	Results, Table 1
Results of individual studies	19	For all outcomes, present, for each study: (a) summary statistics for each group (where appropriate) and (b) an effect estimate and its precision (e.g. confidence/credible interval), ideally using structured tables or plots.	Results
Results of syntheses	20a	For each synthesis, briefly summarise the characteristics and risk of bias among contributing studies.	Results
	20b	Present results of all statistical syntheses conducted. If meta-analysis was done, present for each the summary estimate and its precision (e.g. confidence/credible interval) and measures of statistical heterogeneity. If comparing groups, describe the direction of the effect.	Results
	20c	Present results of all investigations of possible causes of heterogeneity among study results.	Results
	20d	Present results of all sensitivity analyses conducted to assess the robustness of the synthesized results.	Results
Reporting biases	21	Present assessments of risk of bias due to missing results (arising from reporting biases) for each synthesis assessed.	Results
Certainty of evidence	22	Present assessments of certainty (or confidence) in the body of evidence for each outcome assessed.	Results
**DISCUSSION**			
Discussion	23a	Provide a general interpretation of the results in the context of other evidence.	Discussion
	23b	Discuss any limitations of the evidence included in the review.	Discussion
	23c	Discuss any limitations of the review processes used.	Discussion
	23d	Discuss implications of the results for practice, policy, and future research.	Discussion
**OTHER INFORMATION**			
Registration and protocol	24a	Provide registration information for the review, including register name and registration number, or state that the review was not registered.	Methods
	24b	Indicate where the review protocol can be accessed, or state that a protocol was not prepared.	Methods
	24c	Describe and explain any amendments to information provided at registration or in the protocol.	Methods
Support	25	Describe sources of financial or non-financial support for the review, and the role of the funders or sponsors in the review.	Declaration
Competing interests	26	Declare any competing interests of review authors.	Declaration
Availability of data, code and other materials	27	Report which of the following are publicly available and where they can be found: template data collection forms; data extracted from included studies; data used for all analyses; analytic code; any other materials used in the review.	N/A

From: Page MJ, McKenzie JE, Bossuyt PM, Boutron I, Hoffmann TC, Mulrow CD, et al. The PRISMA 2020 statement: an updated guideline for reporting systematic reviews. BMJ 2021;372:n71. doi: 10.1136/bmj.n71.
This work is licensed under CC BY 4.0. To view a copy of this license, visit https://creativecommons.org/licenses/by/4.0/

Systematic Review Screening Tool

Our questions for the review are:

- What processes are put in place to protect the legal rights of people living with dementia who are detained in health and social care settings?
- Who is involved in the decision-making process?
- How are the views of the person with dementia taken into consideration (or not)?
- To what extent are the views of carers considered throughout the detention process?
- What other factors impact on professional decision-making in this context?

Column Number	Ref ID	Citation and DOI	Method	Reviewer 1	Reviewer 2	Themes
[Column numbers to be added as necessary from the screening table].	[Reference IDs to be added as necessary from the screening table].	[Citations and DOIs for each article to be added here].	[Method to be added i.e. quant, qual or mixed methods].	[Name of first reviewer to be added here].	[Name of second reviewer to be added here].	[Themes to be added by each reviewer in free text].
